# Subjects for Discussion at the Next Meeting of the Mississippi Valley Association

**Published:** 1860-02

**Authors:** 


☞ The Executive Committee respectfully submit the following subjects for discussion, at the next meeting of the Mississippi Valley Association of Dentists:

I. Mechanical Dentistry,
Continuous Gum Work,
Vulcanite Base,
Cheoplasty,
Gold Work.

II. What is the best treatment when the pulp of a tooth is exposed, in order to secure its vitality?

III. Filling teeth.

IV. Irregularities of the teeth, their causes and modes of treatment.

V. Structure and nutrition of the Dental Tissues.

VI. Hemorrhage following extraction of the teeth and best treatment.

E. Collins,

L. B. Hatch,

H. A. Smith.

} Committee.

